# The Failure of Voltage Divider Induced by Insulating Material Degradation Under Coupling Effect of High-Frequency Field and Temperature

**DOI:** 10.3390/ma19102047

**Published:** 2026-05-14

**Authors:** Xuan Li, Chuang Zhang, Zixi Liu, Jiajie Song, Huidong Tian, Qijia Xie, Zhengmao Zhang, Shengtao Li

**Affiliations:** 1 Equipment Management Department of State Grid Corporation of China, Beijing 100032, China; phoenixuan@foxmail.com; 2 School of Electrical Engineering, Xi’an Jiaotong University, Xi’an 710049, China; lzx17628068926@163.com; 3 State Grid Economic and Technological Research Institute Co., Ltd., Beijing 102200, China; 4 State Grid Hubei Electric Power Co., Ltd., Wuhan 430048, China

**Keywords:** high-frequency harmonics, failure of divider, polypropylene/paper/polypropylene, dielectric loss, flashover

## Abstract

This paper systematically investigates the failure characteristics and mechanisms of insulating materials in DC voltage dividers under combined high-frequency voltage and high-temperature conditions via simulations and experiments. The results showed that high-frequency harmonics severely degrade the insulation strength of polypropylene/paper/polypropylene (PPLP) at 10 kHz, in which the bulk breakdown strength of PPLP decreases by over 50%. Furthermore, the surface flashover voltage in oil is reduced by 17.7% under high-frequency voltage alone, and by as much as 51% when white flocculent substances are present in the oil. The dielectric properties of PPLP strongly depend on frequency and temperature, which aggravate the heat accumulation of the divider under high-frequency voltage. Furthermore, the multilayer structure of PPLP introduces deeper trap levels due to interfacial states, which reduce the breakdown strength and flashover voltage of PPLP. Electro-thermal coupling induces a rapid temperature rising to 98 °C at 25 kHz caused by dielectric loss, leading to oil turbidity and white precipitation, consistent with finite element simulations. Consequently, a failure mechanism is proposed as follows: prolonged electro-thermal stress causes chain scission in styrene-containing materials, releasing monomers that repolymerize into white polystyrene deposits. Their porous structure and dielectric mismatch distort the interfacial field, trigger partial discharge, and aggravate surface flashover.

## 1. Introduction

**Figure 1:**
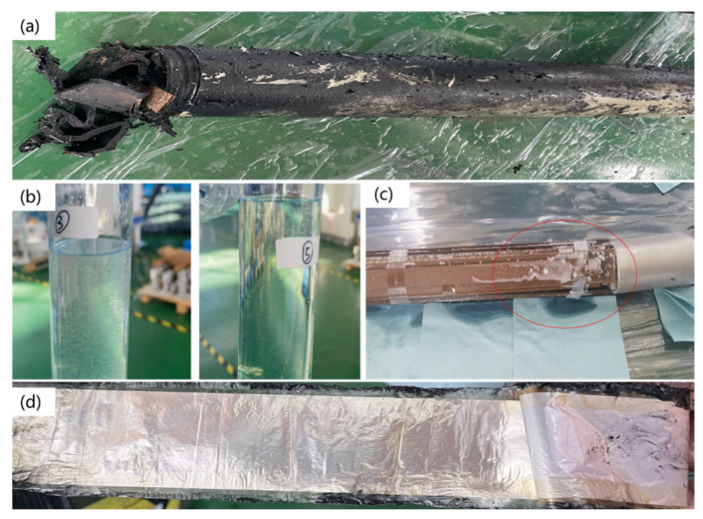
Disassembly diagram of the voltage divider that failed in the practical engineering with (**a**) overall appearance, (**b**) the white substance in the oil extracted from the voltage divider, (**c**) the white substance adhering to the paperboard, and (**d**) the insulating materials with aluminum foil electrode.

In a flexible DC transmission system, the DC voltage divider is a core measurement component [1,2,3]. It is responsible for accurately converting the extremely high DC voltage into signals recognizable by secondary equipment [4,5,6,7]. Its accuracy and reliability, which is influenced by the reliability of insulation materials used dominantly, directly determine the safe operation level of the entire flexible DC grid [8,9]. However, recent failures of DC voltage dividers have occurred, as shown in Figure 1, and high-frequency harmonics of 10 kHz have been detected [10]. The widespread integration of a high proportion of renewable energy sources and power electronic equipment has given rise to notable nonlinear characteristics within the system, resulting in the generation of high-frequency harmonics exceeding 10 kHz. The emergence of such harmonics in HVDC converter valves primarily stems from characteristic sub-harmonics introduced by step-wave switching, as well as non-characteristic sub-harmonics arising from sub-module switching operations. Furthermore, it has been found that a white solid substance precipitates from the oil used in the voltage divider, which may be attributed to the deterioration of polypropylene/paper/polypropylene (PPP), namely polypropylene laminated paper (PPLP), in the voltage divider [11,12].

There have been numerous studies on the insulation failure of materials used in voltage dividers. It is revealed that PPLP exhibits a threshold electric field of DC conductivity as high as 34.4 kV/mm, and PPLP can withstand electric fields up to 80 kV/mm without undergoing breakdown [13]. This superior performance is primarily attributed to the inherently low polarization loss of the polypropylene layer, in combination with charge trapping and reverse electric field shielding effects at the PP/paper interface, which effectively suppress carrier injection [14,15,16]. It is indicated that the breakdown strength of PPLP decreased as the thickness of the individual sheets increased where the average AC breakdown strength of laminated polypropylene paper (LPP) dropped from 67.2 kV/mm to 54.3 kV/mm, while the average lightning impulse breakdown strength decreased from 116.7 kV/mm to 98.1 kV/mm [17]. Experimental results showed that, as the temperature increased to 100 °C, the breakdown field strength of PPLP decreased by 44.5% to 213.9 kV/mm. These findings indicate that PPLP demonstrates superior breakdown performance under elevated temperature conditions [18]. In PPLP insulation, charge accumulation at the interface between polypropylene (PP) and kraft paper plays a critical role in modulating the electric field distribution [19]. After applying a DC electric field of 8 kV/mm for 50 min, the electric field strength within the PP layer increased to approximately 16 kV/mm, which indicates that the behavior of space charge should be considered carefully [20]. Findings indicate that harmonic components superimposed on dc voltage pose a significant threat to the PPLP insulation [21]. However, it was also found that, under DC electric fields up to 30 kV/mm, no space charge accumulation was detected within the bulk of PPLP [22]. Obviously, the existing research focus on the breakdown or space charge behavior of PPLP under DC or low harmonic voltage.

Additionally, some studies concern the flashover performance of PPLP. Experimental results demonstrate that the DC flashover voltage of PPLP perpendicular to the fiber direction is approximately 15% higher than that parallel to the fiber direction [23]. It was also reported that PPLP exhibits the highest inception electric field strength among tested materials, with moderate flashover voltage levels. Furthermore, the optimized surface roughness of PPLP enables a balanced trade-off between high inception voltage and robust flashover resistance, making it particularly suitable for regions with concentrated electric fields [24]. Furthermore, it was confirmed that the DC surface flashover strength of PPLP decreased over 40% due to tensile stress, which indicates that the working environment obviously influences the insulation [25]. It has been demonstrated that the surface flashover voltage and field strength of polypropylene films are positively correlated with the interlayer pressure and negatively correlated with the experiment temperature [26]. In addition, it is indicated that the flashover field strength decreases by 44% and gradually tends to be stable as the clear edge width varies from 0.5 to 2.5 mm [27]. Nevertheless, the flashover of PPLP under high-frequency voltage has not been reported.

In summary, most existing studies have focused on the influence of relatively low-frequency harmonics on the failure characteristics of insulating materials used in DC voltage dividers, which cannot explain the failure mechanism of the voltage divider under high-frequency voltage [9,28,29,30]. Consequently, a physical process describing the insulation failure of DC voltage dividers under high-frequency harmonics has not yet been established, which hinders the improvement of their tolerance to high-frequency harmonics. Furthermore, it is found that harmonic components exceeding 200 kHz undergo severe attenuation during transmission, resulting in amplitudes significantly lower than those of lower-frequency counterparts [31].

As a result, this paper investigates the breakdown characteristics of PPLP under high-frequency harmonic voltages up to 200 kHz, and compares them with those under power frequency voltage. In order to reveal the insulation failure of the voltage divider, the flashover of PPLP under 10 kHz was performed. By characterizing the dielectric properties and trap distribution of the insulating materials, the mechanisms underlying their breakdown and surface discharge are proposed. Using a combination of simulation and experimental methods, the performance degradation and decomposition characteristics of the insulating materials under high-frequency harmonic voltages are studied. Finally, the insulation failure mechanism of DC voltage dividers under high-frequency harmonic voltages, triggered by the deterioration of the insulating materials, is proposed.

## 2. Materials and Methods

### 2.1. Experimental Methods for Voltage Divider and Its Insulating Materials

#### 2.1.1. Breakdown Strength Testing of Insulating Materials

**Figure 2:**
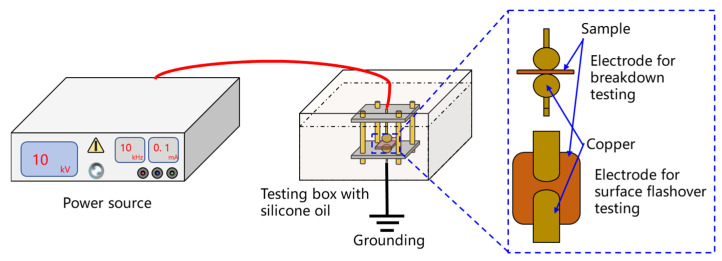
The platform of breakdown field and surface flashover testing of insulating materials in voltage divider.

The platform for breakdown and flashover testing for insulating materials in the voltage divider was built, as shown in Figure 2. A custom built, wide frequency range power supply (50 Hz–30 kHz, 0–50 kV) developed by the research team served as the breakdown source. A sphere–sphere electrode configuration was employed in accordance with IEC 62539 [32]. The three-layer dielectric material of polypropylene/paper/polypropylene with a thickness of 0.036 mm was immersed in transformer oil. The voltage was increased at a rate of 1 kV/s until dielectric breakdown occurred. All breakdown experiments were conducted at room temperature (25 °C). The breakdown field strength was then obtained by dividing the breakdown voltage by the thickness of the dielectric material. The characteristic breakdown strength was statistically determined using the Weibull distribution, with a minimum of 10 samples tested under each experimental condition.

A finger-type electrode configuration with a gap distance of 0.5 mm was used. The voltage was increased at a rate of 1 kV/s until a surface flashover occurred along the dielectric material, and the corresponding voltage was recorded. Surface flashover voltages were measured under power frequency and a representative high-frequency (10 kHz) condition. The influence of fibrous contaminants present in practical operating conditions was also considered. The characteristic flashover strength was statistically determined using the Weibull distribution, with a minimum of 10 tests performed for each experimental condition.

#### 2.1.2. Surface Potential Decay Test

In this study, the isothermal surface potential decay (ISPD) method was employed to characterize the surface potential decay behavior of epoxy resin samples [33]. By fitting the experimental data using a bimodal model, the trap distribution characteristics and charge transport properties within the epoxy resin were obtained, facilitating an understanding of the changes in charge transport behavior during electrical tree aging experiments.

The test system is placed in a sealed environment to ensure that the temperature and humidity remain unaffected by external conditions during the measurement. The system mainly consists of a charging electrode system, an electrostatic voltage sensing system, a data acquisition and conversion system, and a temperature control system. The charging electrode system, based on corona discharge, is used to charge the thin film samples. It employs a needle–grid–plate electrode configuration, comprising a high-voltage needle electrode, a metal grid electrode, and a grounded electrode. The needle electrode is made of stainless steel, the grid is a stainless-steel ring with 1 mm × 1 mm mesh openings, and the grounded electrode is a smoothly polished aluminum plate. The distance between the needle electrode and the grid electrode is 5 cm, and the distance between the grid electrode and the grounded electrode is also 5 cm. The measurement system primarily consists of an electrostatic capacitance probe and a non-contact electrostatic voltmeter. The voltmeter model is Trek P0865(Trek Inc., Lockport, NY, USA), capable of measuring surface potentials ranging from −10 kV to +10 kV, with a probe resolution of 1 V. After the voltmeter records the surface potential data, the computer-based data acquisition and recording system enables continuous sampling and storage of the potential decay process, exporting the data as an Excel spreadsheet.

#### 2.1.3. Dielectric Spectroscopy Test

The dielectric properties of the insulating materials used in the voltage divider were characterized using a broadband dielectric spectrometer. Measurements were performed over a frequency range of 0.1 Hz to 10^6^ Hz and a temperature range of 20 °C to 90 °C. This yielded the temperature- and frequency-dependent characteristics of the dielectric constant and dielectric loss tangent (i.e., the frequency spectra and temperature-dependent spectra) for the insulating materials. The findings from N. M. Chalashkanov et al. demonstrated that sputtered gold electrodes establish near-uniform electrical contacts, effectively eliminating dispersive processes and creating a barrier effect against potential ionic species present in the epoxy resin [34]. This electrode configuration ensures the accurate characterization of intrinsic material properties by mitigating interfacial artifacts and ion-related contributions, thereby enhancing measurement reliability across a wide frequency temperature range. As a result, sputtered gold electrode samples were used in this paper. These data were subsequently incorporated into the simulation model described in Section 2.1.

#### 2.1.4. Temperature Rise Test

**Figure 3:**
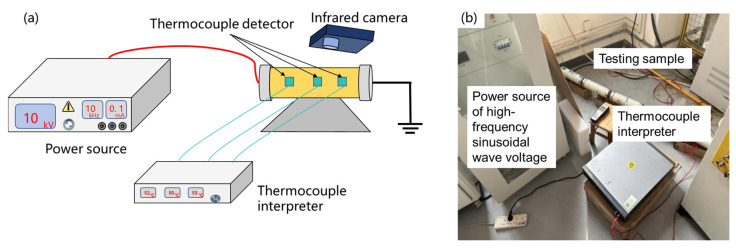
The temperature rising measurement of the core of voltage divider with (**a**) diagram and (**b**) practical testing platform.

A test platform, as shown in Figure 3, was established to investigate the temperature rise in capacitors under high-frequency sinusoidal voltage. The sample under test was formed by connecting five 12,000 pF capacitors in series, resulting in a total capacitance of 2400 pF. A high-frequency sinusoidal power supply with a high frequency of 25 kHz was used to energize the test sample, and temperature rise tests were carried out at 9.8 kV, 8.7 kV, and 5 kV. An ammeter was connected in series to monitor and determine the circuit current, and an infrared camera and thermocouple patch thermometers were employed for temperature monitoring. The four measurement points for the thermocouple thermometers were sequentially located at the beginning, middle, and end of the first capacitor on the high-voltage end, as well as at the end of the last capacitor.

### 2.2. Simulation of Temperature Rise in the Core of Voltage Divider

Under high-frequency voltages, the main heat source in a voltage divider is the dielectric loss heating. As shown in Equation (1), the dielectric loss heating depends primarily on the frequency and electric field strength of the applied voltage, as well as the dielectric constant and dielectric loss tangent of the material. Since the dielectric constant and loss tangent of the insulating material are functions of voltage frequency and temperature, the heating process in the voltage divider constitutes an electro-thermal coupled process.

**Figure 4:**
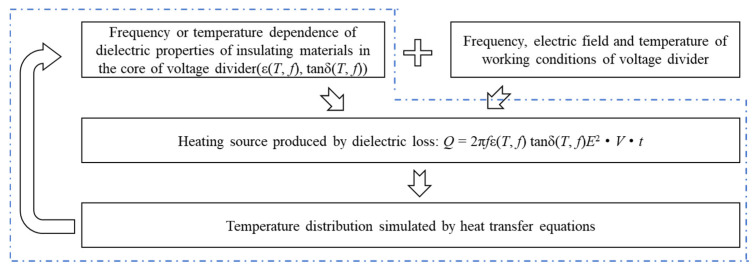
The diagram of simulating methods of temperature in the core of voltage divider caused by dielectric loss.

We propose a time-dependent calculation procedure for dielectric loss heating in voltage dividers under high-frequency voltage, as illustrated in Figure 4. The heat transfer equations are given by Equation (2). The dependence of the dielectric constant and dielectric loss tangent of the insulating material on voltage frequency and temperature is determined from the dielectric frequency spectra and temperature-dependent spectra described in Section 2.2.

**Figure 5:**
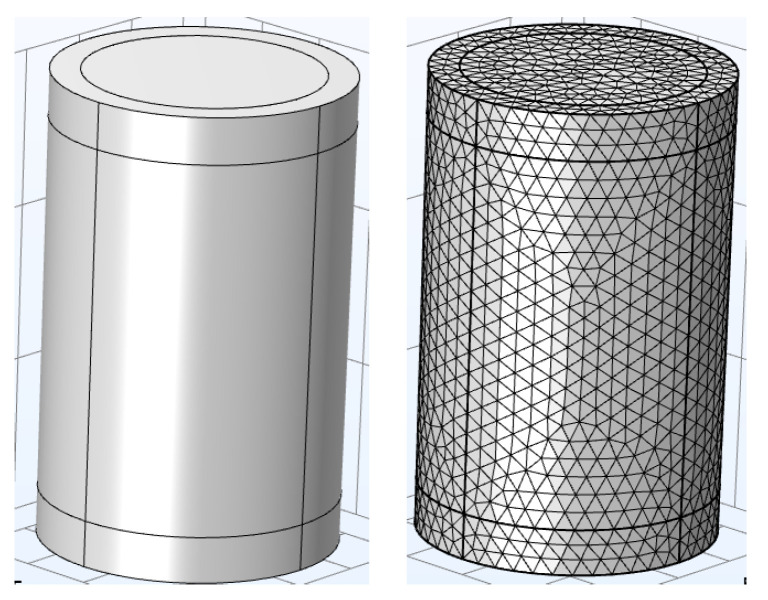
Simulation model of the capacitor in voltage divider.

**Figure 6:**
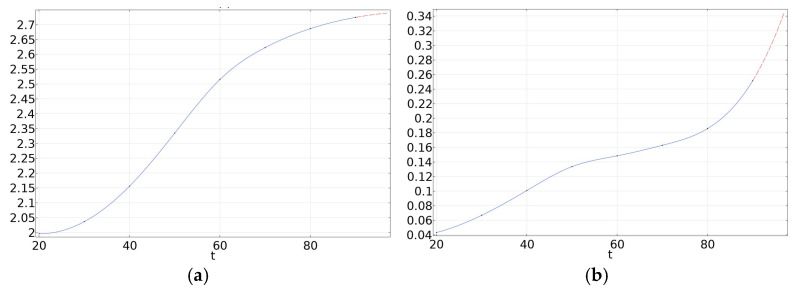
Parameters used in the simulation. (**a**) Temperature dependence of relative dielectric constant; (**b**) temperature dependence of tan *δ*.

*Q* = 2π*f*ε(*T*, *f*) tan δ(*T*, *f*)*E*^2^·*V*·*t* (1)

(2) $$\rho c_{p}\frac{\partial T}{\partial t}=\nabla \cdot (\lambda \nabla T)+Q+q_{\mathrm{rad}}$$

where *Q*, *f*, *T*, *ε*(*T*, *f*), tan δ(*T*, *f*), *E*, *V*, *t*, ρ, *λ*, $q_{\mathrm{rad}}$ are heating source, voltage frequency, temperature, dielectric constant, electric field, volume, time, density, thermal conductivity, heating loss through radiation, respectively. The simulation was performed in Comsol Multiphysics 6.3, and the simulating models are shown in Figure 5. The heat source was defined as the dielectric loss that can be obtained according to the temperature dependence of the relative dielectric constant and tan δ, as illustrated in Figure 6. The applied voltage and ambient temperature were set according to the experiment in Section 2.1.4.

## 3. Results and Discussions

### 3.1. The Breakdown and Flashover of Insulating Materials

**Figure 7:**
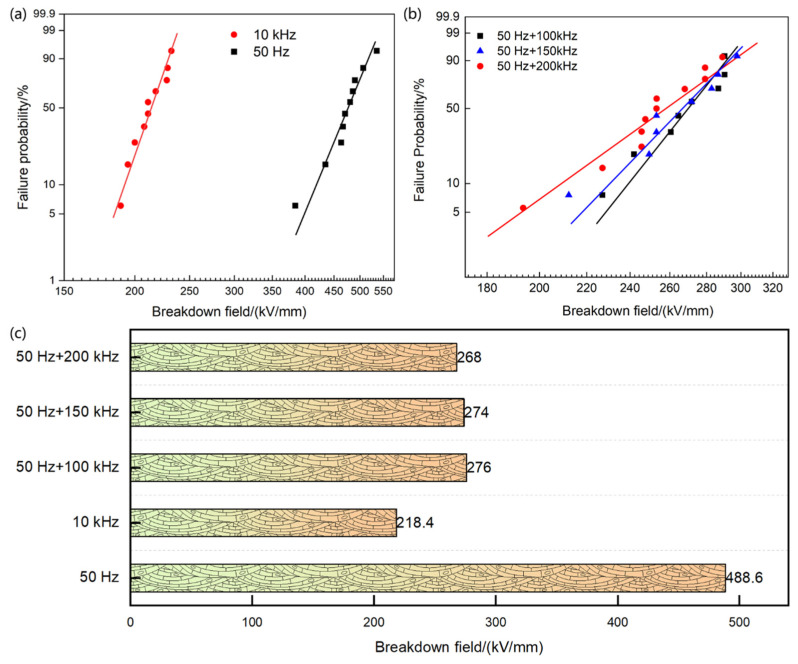
The breakdown field of PP/PA/PP at varied voltage waveforms with (**a**) Weibull distribution of breakdown field under 50 Hz and 10 kHz, (**b**) Weibull distribution of breakdown field under 50 Hz + 100/150/200 kHz, (**c**) typical breakdown field under varied voltage waveforms.

The breakdown strength of the insulating material used in the voltage divider under power frequency and high-frequency voltages is shown in the figure below. To investigate whether the insulation failure of the voltage divider is caused by a bulk breakdown of the insulating material, we also considered an extreme condition involving power frequency voltage distortion, specifically the influence of power frequency voltage superimposed with high-frequency harmonics above 100 kHz, as shown in Figure 7. The results indicate that the breakdown strengths of the insulating material under power frequency, 10 kHz, power frequency superimposed with 100 kHz, power frequency superimposed with 150 kHz, and power frequency superimposed with 200 kHz are 488.6, 218.4, 276, 274, and 268 kV/mm, respectively. Compared with power frequency, the breakdown strength under high-frequency harmonic voltages decreased by 55.3%, 43.5%, 43.9%, and 45.1%, all of which are significantly lower than the service field strength of the insulating material in practical engineering scenarios. Furthermore, we observed that the breakdown strength of the insulating material decreased the most under a single high-frequency harmonic voltage. Therefore, in the following sections, we will focus on the effects of power frequency and single high-frequency harmonics.

Since the bulk breakdown strength is significantly higher than the operating field strength of the voltage divider in service, and existing studies have shown that the interfacial breakdown strength of materials is often lower than the bulk breakdown strength [35,36,37,38], we investigated the surface flashover strength of the insulating material used in the voltage divider in oil. Considering the potential influence of the white flocculent substance, we tested the power frequency and high-frequency surface flashover strength of the polypropylene laminated paper used in the voltage divider, both with and without the flocculent substance present in the insulating oil.

**Figure 8:**
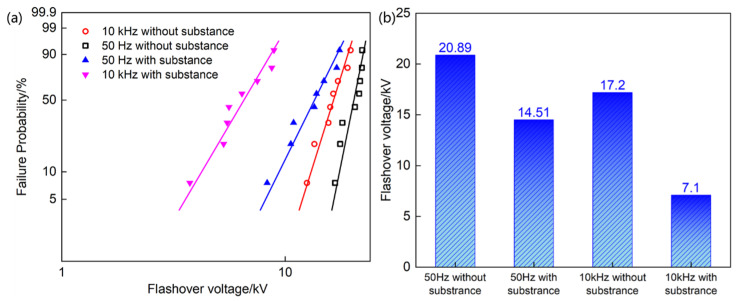
The breakdown field at the interface of oil/PP under 50 Hz and 10 kHz with (**a**) Weibull distribution and (**b**) typical values.

The results in Figure 8 indicate that, under the same experimental conditions, the high-frequency surface flashover strength is considerably lower than the power frequency surface flashover strength, and the presence of the flocculent substance further reduces the breakdown strength of the insulating material. Specifically, in the absence of the flocculent substance, the power frequency and high-frequency surface flashover voltages were 20.89 kV and 17.2 kV, respectively, with the high-frequency value being 17.7% lower than the power frequency value. In the presence of the flocculent substance, the power frequency and high-frequency surface flashover strengths were 14.51 kV and 7.1 kV, respectively, with the high-frequency value being 51% lower than the power frequency value. Consequently, the presence of the flocculent substance reduced the power frequency and high-frequency surface flashover strengths by 30.5% and 58.7%, respectively, leading to a nearly 66% decrease under high-frequency conditions compared with power frequency conditions.

### 3.2. The Dielectric Properties and Trap Characteristics of Insulating Materials

The complex permittivity $\tilde{\varepsilon }(\omega )=\varepsilon^{\prime }(\omega )-j\varepsilon^{\prime \prime }(\omega )$ is a complex-valued frequency-domain constitutive parameter describing the polarization response of a dielectric medium to an alternating electric field. It is defined by the relation $\tilde{D}=\tilde{\varepsilon }\tilde{E}$, where $\tilde{D}$ is the electric displacement and $\tilde{E}$ the electric field strength. The real part *ε*′ characterizes the ability of the dielectric to store electric field energy, representing the elastic component of the polarization process; it determines the permittivity and the real part of the refractive index of the material. The imaginary part *ε*″ quantifies the dissipation of electric field energy into heat, originating from the damping process in which polarization lags behind the electric field, corresponding to dielectric loss. The ratio $tan\;\delta =\varepsilon^{\prime \prime }/\varepsilon^{\prime }$ is defined as the loss tangent, which measures the relative energy loss per unit cycle.

**Figure 9:**
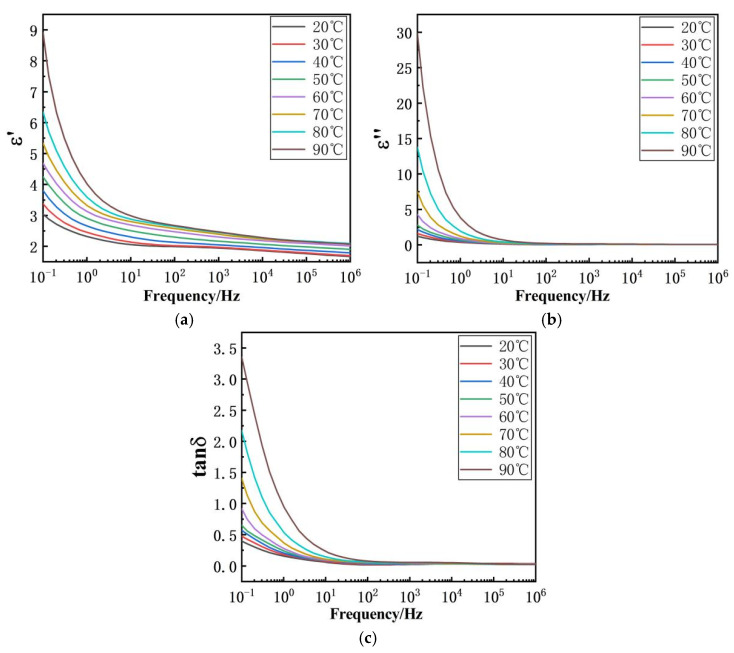
The frequency and temperature dependence of (**a**) real part of the relative dielectric constant, (**b**) imaginary part of the relative dielectric constant, and (**c**) dielectric loss tangent of insulating materials in voltage divider.

This study investigates the dielectric properties of oil-immersed capacitor film using dielectric spectroscopy, which is shown in Figure 9. The results show that the real part of the complex permittivity, *ε*′, decreases with increasing frequency, and the rate of decline gradually slows as frequency rises. Additionally, *ε*′ increases with temperature. Specifically, at 20 °C, *ε*′ of the oil-immersed capacitor film is 3.0385 at 0.1 Hz, 1.9901 at 89.105 Hz, and 1.6715 at 1 MHz. At 50 °C, *ε*′ is 4.2489 at 0.1 Hz, 2.3045 at 89.105 Hz, and 1.902 at 1 MHz. At 90 °C, *ε*′ reaches 8.9105 at 0.1 Hz, 2.1352 at 89.105 Hz, and 2.0707 at 1 MHz.

The real part of the permittivity characterizes the material’s ability to store electrical energy. In the low-frequency range (10^−1^–10^2^ Hz), the period of the applied electric field is much longer than the characteristic time required for relaxation polarizations to establish. Under such conditions, orientational and interfacial polarizations can fully develop in response to the field, leading to a higher ε′ at low frequencies. As the frequency increases, the field period shortens; when it becomes shorter than the relaxation time, these polarization mechanisms can no longer follow the field variations completely, and their contribution to ε′ diminishes. Consequently, ε′ decreases monotonically with increasing frequency. Temperature elevation enhances the thermal motion of molecules, which facilitates the occurrence of orientational and interfacial polarization and thereby increases the polarization intensity. At the same time, higher temperatures reduce the relaxation time, enabling relaxation polarizations to establish more readily within a shorter field period. Therefore, within a certain temperature and frequency range, an increase in temperature promotes the full development of relaxation polarizations, resulting in an increase in ε′.

The imaginary part of the complex permittivity, ε″, and the dielectric loss tangent, tan δ, both decrease with increasing frequency and eventually approach constant values, while increasing with rising temperature. Specifically, at 20 °C, ε″ of the oil-immersed capacitor film is 1.2096 at 0.1 Hz, 0.03688 at 89.105 Hz, and 0.0543 at 1 MHz. At 50 °C, ε″ is 2.7743 at 0.1 Hz, 0.1191 at 89.105 Hz, and 0.04896 at 1 MHz. At 90 °C, ε″ reaches 29.869 at 0.1 Hz, 2.6825 at 89.105 Hz, and 0.0485 at 1 MHz.

Dielectric loss within the material is closely related to its internal heat generation. The imaginary part of the permittivity (ε″) represents the active loss per unit volume within the dielectric, and its variation with frequency generally follows the same trend as the loss tangent (tan δ). In the low-frequency range (10^−1^–10^2^ Hz), the polarization intensity within the material is substantial, allowing relaxation polarization to fully develop. Consequently, the associated energy dissipation during polarization is correspondingly high. As the frequency increases, relaxation polarization gradually fails to keep pace with the alternating electric field, leading to a reduction in the loss component associated with this mechanism. Thus, tan δ exhibits a decreasing trend with increasing frequency. When the frequency increases further into the high-frequency range, the period of the electric field becomes extremely short. Under such conditions, conduction losses become negligible, and relaxation polarization can no longer establish effectively. Only instantaneous displacement polarization persists within the dielectric. At this point, both the loss tangent and the imaginary part of the permittivity gradually stabilize and approach relatively low constant values.

To investigate the variations in the trap energy level and density of polypropylene before and after aging, both aged and unaged polypropylene films were employed in this study. The surface potential decay (SPD) technique was adopted to characterize the trapping properties of the material. Previous studies have demonstrated that the SPD method enables the unique determination of the relaxation time constant from the experimental decay curve without relying on the specific form of the decay function, thereby providing a reliable characterization of trap-controlled charge transport behavior [37].

**Figure 10:**
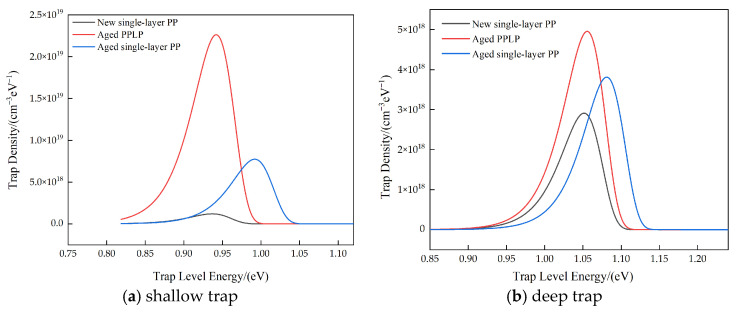
Test results for surface potential decay of aged polypropylene laminated paper and pure polypropylene.

Subsequent studies performed surface potential decay measurements on aged PPLP and pure polypropylene, as illustrated in Figure 10. The results indicate that the three-layer structure of polypropylene composite paper (polypropylene/insulating paper/polypropylene) exhibits considerably higher trap energy levels and trap density compared with single-layer polypropylene. This enhancement is primarily attributed to the synergistic effect between deep-level defects introduced by heterointerfaces and intrinsic bulk traps within the material. As a porous fibrous medium, the insulating paper not only contributes abundant intrinsic deep traps with energy levels exceeding 1.0 eV through its extensive cellulose-based interfaces and porous architecture but also induces lattice mismatch and band bending at the heterointerface with the polypropylene layer. Such interfacial mismatches result in a high density of interface states, which typically feature deeper energy levels than those of the bulk phase of a single material, thereby enhancing charge capture efficiency. Moreover, the multilayer configuration restricts charge carrier transport along the thickness direction, thereby increasing the probability of charge trapping. Macroscopically, this manifests as a slower surface potential decay rate, leading to higher apparent trap energy levels and trap densities when estimated via inversion based on the isothermal relaxation current model. Furthermore, the trap characteristics of aged polypropylene is conducted and added, indicating that aged polypropylene performs at deeper energy depths. This leads to the decrease in the surface insulation of PPLP.

### 3.3. Analysis of XRD Testing Results

**Figure 11:**
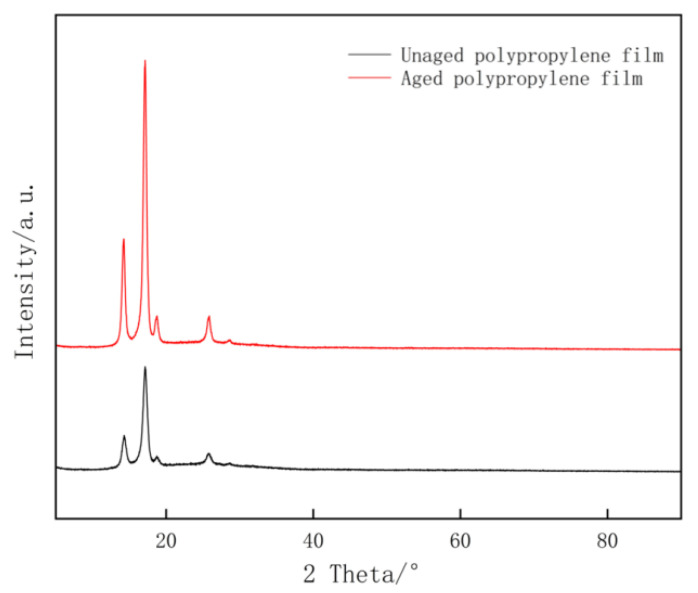
XRD test results for polypropylene film before and after aging.

Figure 11 presents the X-ray diffraction (XRD) patterns of the polypropylene film before and after the aging process. The diffraction profiles exhibit characteristic peaks corresponding to the α -monoclinic crystal phase of isotactic polypropylene. Specifically, four distinct diffraction peaks are observed at 2θ angles of approximately 14.1°, 16.9°, 18.6°, and 21.1°, which correspond to the (110), (040), (130), and (111) crystallographic planes, respectively. A comparative analysis reveals a significant evolution in the crystalline structure induced by aging. While the peak positions remain virtually unchanged—indicating that the lattice parameters and unit cell dimensions are preserved—the diffraction intensities of all characteristic planes, particularly the dominant (110) and (111) peaks, are markedly enhanced in the aged sample compared with the pristine sample. This phenomenon can be attributed to the mechanism of physical aging and secondary crystallization. During the aging process, the polymer chains in the amorphous regions undergo structural relaxation and reorganization. Driven by thermodynamic forces to minimize free energy, the disordered chain segments migrate towards the crystal–amorphous interface and incorporate into the existing lamellae. This results in lamellar thickening and an increase in the overall degree of crystallinity. Consequently, the enhanced intensity of the (110) and (111) reflections confirms that the aging treatment promotes the perfection of the crystal structure and the growth of crystalline domains within the PP film.

### 3.4. The Spatial and Temporal Distribution of Electric Field and Temperature in the Core of Voltage Divider

#### 3.4.1. The Experimental Results

**Figure 12:**
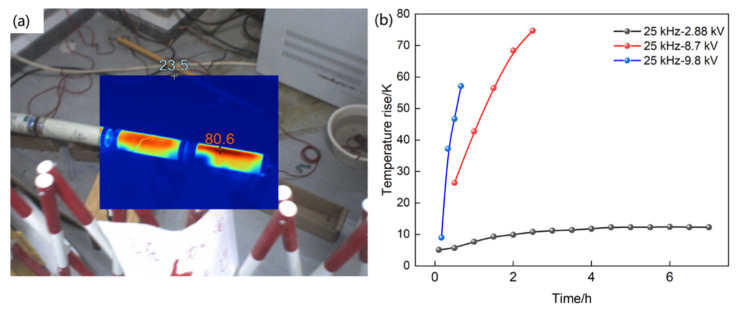
(**a**) Experimental spatial and (**b**) experimental temporal distribution of temperature in the core of voltage divider at 25 kHz.

To further analyze the degradation characteristics of the capacitor insulation, an accelerated aging test was conducted by applying a higher current to the capacitor. After the capacitor was subjected to 2.88 kV for 8 h, 9.8 kV for 40 min, 8.7 kV for 2 h, and 4.6 kV for 90 h, the capacitor oil became visibly turbid. The experimental results are depicted in Figure 12.

When a voltage of 8.7 kV at 25 kHz with a current of 2983 mA was applied for 2 h, the surface temperature reached 98 °C, corresponding to a surface temperature rise of approximately 75 K. Under stable conditions at 4.6 kV, 25 kHz, and 1715 mA, the surface temperature reached 80 °C, with a surface temperature rise of about 55 K. Due to the high test current and rapid temperature increase, the experimental conditions did not permit further temperature escalation. However, the observed phenomena clearly indicate that a high-current environment with multiple harmonics can cause a significant temperature rise in the capacitor, leading to the precipitation of a substantial amount of white substance.

#### 3.4.2. The Simulated Results

**Figure 13:**
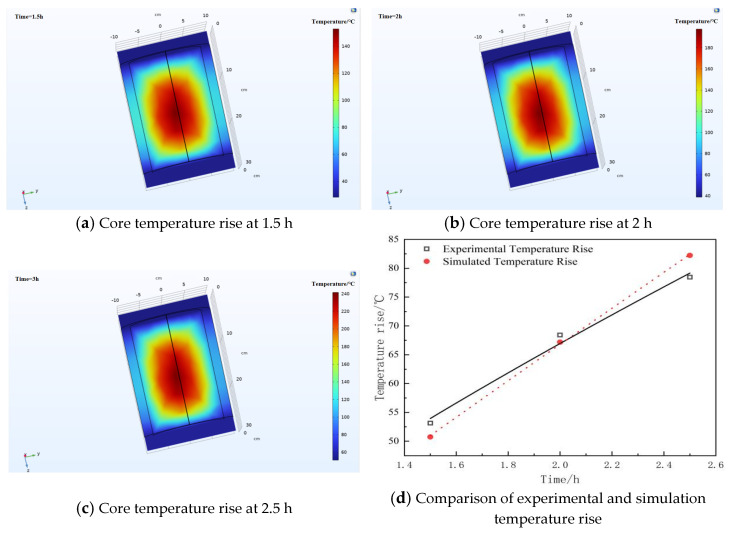
Simulated distribution of voltage divider core temperature at different times under sinusoidal wave voltage of 8.7 kV, 25 kHz conditions.

Subsequently, a finite element simulation was conducted to validate the temperature rise experiment using the COMSOL Multiphysics platform with version of 6.3. A simulation model of the voltage divider was established within the software under the same conditions as those in the experimental temperature rise test; specifically, a voltage with a frequency of 25 kHz and an amplitude of 8.7 kV was applied to the divider. The simulation calculated the temperature rise of the voltage divider over a duration of 2.5 h, as shown in Figure 13. The results indicate that the temperature of the voltage divider increased by 50.743 K after 1.5 h of voltage application, 67.188 K after 2 h, and 82.23 K after 2.5 h. These simulation results are in good agreement with the findings of the temperature rise experiment.

It was found that measured temperature rises in experiments at 1.5 h, 2 h, and 2.5 h were 47.5 K, 67.3 K, and 76.2 K, respectively. As a result, there were corresponding discrepancies of 6.8%, 0.02%, and 7.92% between the experimental and simulated values. It is obvious that these simulation results are in good agreement with the findings of the temperature rise in experiment.

### 3.5. The Failure Mechanism of Voltage Divider Induced by Insulating Material Degradation Under High Frequency Voltage

**Figure 14:**
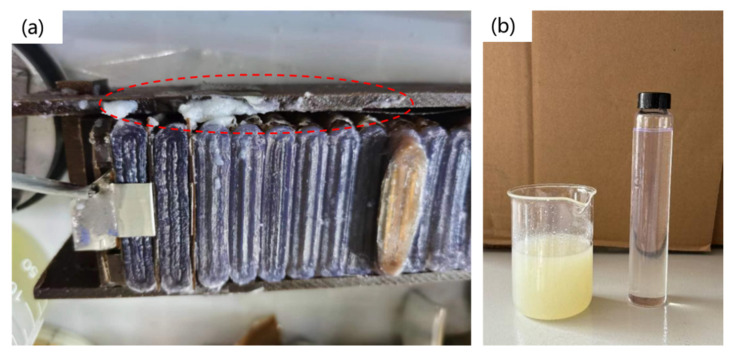
State of insulating material in the voltage divider after high-frequency voltage aging. (**a**) Erosion at the edge of the capacitor core; (**b**) the oil becomes turbid.

In the aforementioned study, long-term aging tests were conducted on capacitors by applying voltages of 2.88 kV for 8 h, 9.8 kV for 40 min, and 8.7 kV for 2 h. Experiments revealed that the capacitor core had been eroded, with a large amount of white substance precipitating on its surface, as shown in Figure 14a that is marked by dashed box. Moreover, after electrical aging, the insulating oil exhibited a distinct change, transitioning from a colorless and transparent state prior to testing to a pale yellow, turbid appearance, as illustrated in Figure 14b.

**Figure 15:**
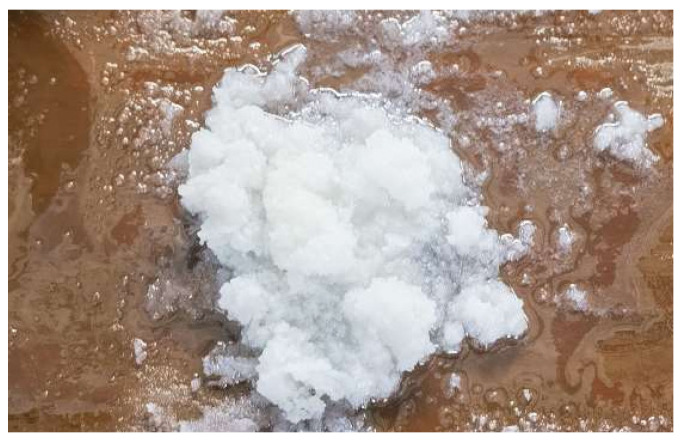
Sampling of deposits on capacitor core surface.

**Figure 16:**
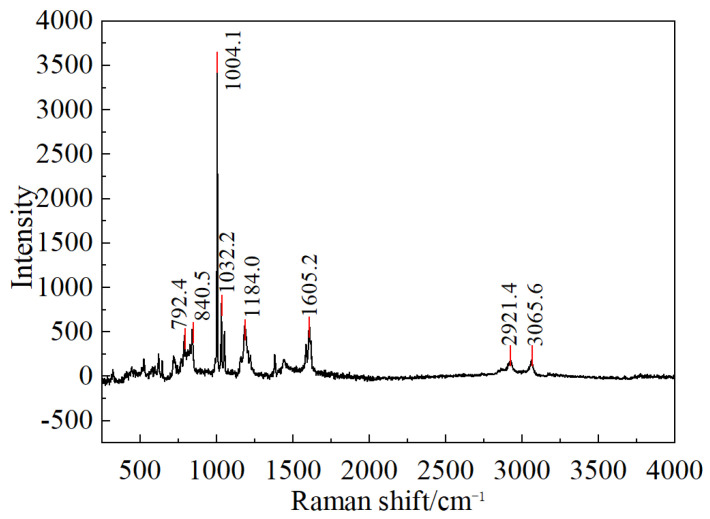
Raman spectral analysis results of white fluffy substance.

Upon completion of operational testing, disassembly of the transformer housing revealed the presence of white flocculent material inside, accompanied by turbidity of the insulating oil. Separation of the insulating oil and the white flocculent material was carried out via vacuum filtration, yielding white crystalline particles retained on the filter paper, as shown in Figure 15. The flocculent material was initially hypothesized to originate from either the insulating oil or the capacitor core. To identify its source, both the white flocculent material and the insulating oil were subjected to Raman spectroscopy and gas chromatography analysis. The Raman spectroscopy results, presented in Figure 16, exhibited a spectral profile highly consistent with the characteristic peaks of polystyrene following database comparison, indicating the highest similarity. The gas chromatography results are summarized in Table 1. Subsequent analysis using the three-ratio method for dissolved gas concentrations suggested that the potential fault types involve electrical discharge and severe overheating.

The formation of white flocculent polystyrene particles within the voltage divider is attributed to the presence of styrene-containing insulating materials used during manufacturing or encapsulation. Under prolonged service conditions, the device is continuously subjected to combined electrical, thermal, and mechanical stresses. These stresses induce polymer chain scission and depolymerization, releasing low-molecular-weight styrene monomers. Driven by electric field migration and concentration gradients, the monomers diffuse toward the core surface, where they undergo secondary polymerization, ultimately accumulating as white flocculent polystyrene deposits.

**Figure 17:**
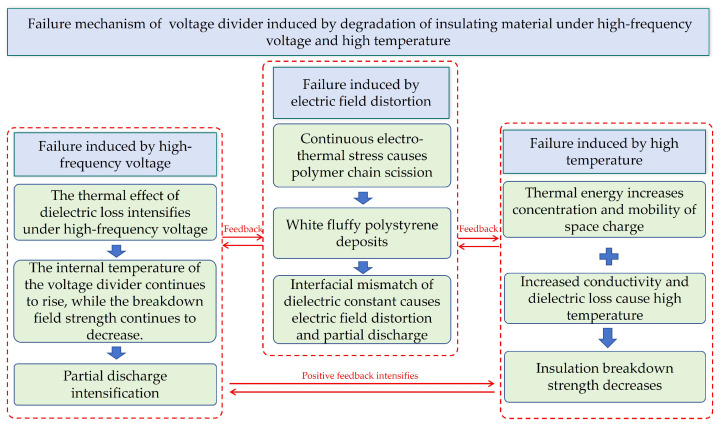
The diagram of failure mechanism of voltage divider under coupling effect of high-frequency voltage and temperature.

Resultingly, this paper proposed the failure mechanism of DC divider induced by the degradation of corresponding insulating materials, as shown in Figure 17. Although the polystyrene deposits are inherently insulating, their loose and porous microstructure makes them highly hygroscopic. In humid environments, moisture absorption forms an electrolytic film on the deposit surface, substantially reducing the surface insulation resistance. Furthermore, the dielectric mismatch between polystyrene and the primary insulation distorts the interfacial electric field, leading to localized field enhancement that can initiate partial discharge (PD). Joule heating from leakage currents on the moist surface promotes alternating dry and wet bands, thereby inducing intermittent spark discharges. While the elevated temperatures from such discharges are insufficient to carbonize polystyrene directly, they thermally decompose the underlying epoxy resin matrix, resulting in the deposition of irreversible conductive carbon residues. Over time, these carbonaceous deposits accumulate and propagate along the electric field direction, eventually forming a permanent semiconductive pathway bridging the high-voltage and grounded terminals. Under overvoltage conditions, this pathway facilitates surface flashovers, culminating in complete insulation failure.

## 4. Conclusions

To clarify the ambiguous breakdown behavior of voltage dividers, this paper systematically investigates the failure characteristics and mechanisms of insulating materials under combined high-frequency voltage and high-temperature conditions through both simulations and experiments. The key findings are as follows:

(1) High-frequency harmonic voltages significantly degrade the insulation strength of materials used in DC voltage dividers. Compared with power frequency conditions, the bulk breakdown strength of polypropylene laminated paper decreases by over 50% under a 10 kHz high-frequency voltage. Furthermore, the surface flashover voltage in oil is reduced by 17.7% under high-frequency voltage alone, and by as much as 51% when white flocculent substances are present in the oil.
(2) Due to the increase in dielectric constant and dielectric loss with rising temperatures at a fixed voltage frequency, the temperature of the insulating material used in the voltage divider continues to rise under high-frequency voltage because of the mutual feedback cycle between the increased dielectric loss and temperature. This leads to changes in the crystalline characteristics of polypropylene, which in turn results in variations in its trap properties. As a result, the traps of materials in the voltage divider exhibit substantially deeper trap energy levels and higher trap densities, enhancing charge capture and hindering carrier transport, which reduce the insulation properties.
(3) Electro-thermal coupling induces a considerable temperature rise and material degradation in the voltage divider core. Under a 25 kHz high-frequency voltage, the capacitor core temperature rises rapidly because of dielectric loss, reaching up to 98 °C, which results in oil turbidity and the precipitation of white substances. Finite element simulations of the temperature rise are in good agreement with experimental measurements, confirming that dielectric loss is the primary heat source.
(4) A failure mechanism for voltage dividers under high-frequency voltage and temperature coupling is proposed. Prolonged electro-thermal stress induces polymer chain scission in styrene-containing materials, releasing monomers that migrate and repolymerize into white polystyrene deposits on the core surface. Owing to its porous structure and dielectric mismatch with the base insulation, this deposit causes interfacial field distortion, triggers partial discharge, and aggravates surface flashover.

## Figures and Tables

**Table 1:** Gas chromatography test results for insulating oil in voltage divider.

Component	CH_4_	C_2_H_4_	C_2_H_6_	C_2_H_2_	CO	CO_2_	H_2_
Concentration	76.21	165.86	249.53	9.33	0	1908.52	9.93

## Data Availability

The original contributions presented in this study are included in the article. Further inquiries can be directed to the corresponding authors.
